# Specific and shared cognitive predictors of drawing and block building in typically developing children

**DOI:** 10.3389/fnhum.2024.1436362

**Published:** 2024-09-25

**Authors:** Isa Zappullo, Vincenzo Paolo Senese, Luigi Trojano, Roberta Cecere, Massimiliano Conson

**Affiliations:** ^1^Developmental Neuropsychology Laboratory, Department of Psychology, University of Campania Luigi Vanvitelli, Caserta, Italy; ^2^Psychometric Laboratory, Department of Psychology, University of Campania Luigi Vanvitelli, Caserta, Italy; ^3^Neuropsychology Laboratory, Department of Psychology, University of Campania Luigi Vanvitelli, Caserta, Italy

**Keywords:** spatial construction, path analysis, cognitive predictors, drawing, block building

## Abstract

**Introduction:**

Spatial construction is a complex ability involving attention, global/local visual processing, mental representation, visuo-motor coordination and, to varying extent, working memory and executive functions, and verbal abilities. In developmental neuropsychology, little attention has been paid to comprehend whether and to what extent the above cognitive processes are involved in two main spatial construction tasks, that is drawing and block building.

**Method:**

We used path analysis to test shared and specific effects of verbal and spatial working memory, spatial attention, inhibition, verbal abilities (vocabulary and naming), figure disembedding, mental rotation, and visual-motor coordination, as well as of demographics (sex, age and socio-economic status), on two classical drawing (Rey-Osterrieth Complex Figure; ROCF) and block building (Block design; BD) tasks in a sample of 195 typically developing children (age range: 7–11 years).

**Results:**

Figure disembedding and visuo-motor coordination were the only shared predictors of both spatial construction tasks. Moreover, ROCF score was directly related with spatial attention and inhibition, while BD score was directly related with sex, vocabulary, mental rotation and backward spatial working memory.

**Discussion:**

These findings distinguish between abilities involved in spatial construction regardless of the type of task and those specifically related to ROCF or Block Design, thus providing clues relevant to neuropsychological assessment and intervention in children with spatial construction disorders.

## Introduction

Spatial construction refers to the ability to reproduce the parts of an array and organize them into an integrated configuration (Stiles et al., 2013, 2020). In clinical practice, it can be classically assessed by a wide range of copying tasks, such as drawing and blocks building (Trojano, 2020; Gainotti and Trojano, 2018; Trojano and Conson, 2008; Stiles et al., 2013, 2020). The most known drawing task is the Rey-Osterrieth Complex Figure (ROCF; Osterrieth, 1944; Rey, 1941) requiring reproducing a complex, multi-part figure as accurately as possible (Lezak, 1995; Shin et al., 2006; Spreen and Strauss, 1998), whereas the most known block building task is the Block Design from the Wechsler scales (Wechsler, 2004) requiring participants to reproduce a model by assembling red and white blocks within a time limit.

In adult neuropsychology, understanding of spatial construction was mainly based on studies considering drawing tasks, in particular copying figures (Van Sommers, 1989; Angelini and Grossi, 1993). Successful execution of copying drawings would be guaranteed by effective visual-perceptual and mental representational (e.g., mental rotation) abilities, working in concert with attention, planning and visual-motor coordination (Angelini and Grossi, 1993; Grossi and Trojano, 1999; Guérin et al., 1999; De Renzi, 1982; Trojano and Conson, 2008).

In the developmental neuropsychological literature, studies on typically developing children converged in showing that drawing is mainly related to visual perception, mental representational and visual-motor coordination, while contrasting data are available on the role of verbal abilities, executive functions and working memory, especially when considering the important changes occurring in spatial construction abilities across developmental ages (Anderson et al., 2001; La Femina et al., 2009; Martens et al., 2014; Morra and Panesi, 2017; Riggs et al., 2013; Weber et al., 2013; Senese et al., 2015, 2020; Smith and Zahka, 2006; Smith et al., 2016; Stiles et al., 2013, 2020; Toomela, 2002). For instance, increased spatial working memory capacity in toddlers seems associated with the transition from scribbling to drawing (Morra and Panesi, 2017). During kindergarten, the tendency of typically developing children up to the age of four to either draw close to or overlap the model has been associated with immature attentional inhibition (Gainotti, 1972; Ambron et al., 2010). Across the school-age years, maturation of visual-motor integration and fine motor control particularly affects drawing speed (Lin et al., 2015). Also, development of visuospatial abilities appears to provide an important contribution to spatial construction at this age period (Del Giudice et al., 2000). Furthermore, global/local visual processing, i.e., the ability to integrate details into a global configuration, progresses considerably from the age of 6 onwards, with significant maturation occurring from 7 to 11 years (Anderson et al., 2001; Martens et al., 2014; Poirel et al., 2011; Scherf et al., 2009), making school age a crucial time window for the study of spatial construction.

Thus, a large literature demonstrates that a wide range of cognitive abilities is involved in the successful execution of copying tasks. Instead, scarce attention has been paid to similarities and differences in the neuropsychological underpinnings of the two main copying tasks, i.e., drawing and block building tasks. Over time the use of drawing tasks has gradually become the most common approach to assess spatial construction in clinical settings (Trojano, 2020), implicitly implying an almost complete overlap between drawing and block building. However, caution is needed in assuming a complete correspondence between the two tasks in the context of neuropsychological assessment since differences have been observed both in adults’ and children’s performance (Gainotti and Trojano, 2018; Trojano, 2020; De Lucia et al., 2019; Martins-Rodrigues et al., 2021; Studeny et al., 2019; Drake, 2014; Conson et al., 2023). Underestimating differences in the cognitive correlates of the two spatial construction tasks could lead to interpretative errors in clinical practice (Studeny et al., 2019; Peart and Bennett, 2024). For this reason, identifying shared and specific predictors of drawing and block building could be relevant for defining tools of neuropsychological assessment.

Recently Zappullo et al. (2021) aimed at looking for similarities and differences in their neuropsychological predictors of ROCF and the Block Design investigating the effect of verbal abilities (naming and verbal knowledge), executive functions (inhibition), figure disembedding and mental rotation on the two tests. The results showed that ROCF copying was predicted by age and figure disembedding, and mediated by inhibition, naming and verbal knowledge, whereas Block Design was predicted by verbal knowledge, figure disembedding and mental rotation and mediated by inhibition and naming. Relevantly, however, an effect of participants’ age was specific for the ROCF copying, leaving unaffected Block Design, thus implying that variables other than those included in the model could exert a specific effect of drawing performance. Indeed, Zappullo et al.’s (2021) study did not consider spatial attention, working memory and visual-motor coordination, that were previously found to be associated with drawing (Senese et al., 2015, 2020).

In the present study, we aimed at bridging this gap by testing the potential effect of previously unconsidered neuropsychological variables on ROCF copying and Block Design to clarify similarities and differences in the cognitive predictors of these two important spatial construction tests. To this aim, we used path analysis and built a model allowing to test, together with variables already considered previously (Zappullo et al., 2021), the contribution of spatial attention, verbal and spatial working memory (keeping separate the passive and active components; Senese et al., 2020), and visual-motor coordination to the two spatial construction tests.

As recalled above, literature indicates that spatial attention (Senese et al., 2020; Russell et al., 2010) and visual-motor coordination (Weber et al., 2013; Lin et al., 2015; Senese et al., 2020) play a role in drawing, but how they interact with each other and with working memory is yet to be fully elucidated. Studies on adult drawing suggested that visual-motor coordination could play a relevant role to support activation copying strategies requiring continuous hand movements under control of back-and-forth eye movements enabling both acquisition of visual information and guidance of the hand on the paper; such a strategy would minimize the effort of spatial working memory (Chamberlain and Wagemans, 2016; Tchalenko and Miall, 2009; Tchalenko et al., 2014). In developmental literature, relationships between spatial attention, visual-motor coordination and working memory can be inferred from data on drawing performance of individuals with Williams Syndrome (WS). Indeed, compared to controls, individuals with WS tend to look less frequently to the model while copying a figure, exhibiting a failure of spatial attention which would result, in turn, in a greater working memory load contributing to impaired drawing performance (Hudson and Farran, 2013). As far as Block Design is concerned, literature on block building tasks found significant contributions of spatial working memory (Ballard et al., 1992, 1997; Nath and Szücs, 2014; Rahbari and Vaillancourt, 2015), as well as of fine motor skills (Jeon et al., 2013). Taken together, in addition to the effects already found in literature (Zappullo et al., 2021), visual-motor coordination is expected to be a shared predictor of the two spatial construction tests, whereas spatial attention and working memory could represent task-specific predictors.

## Methods

### Participants

To identify a proper target sample size, we conducted an *a priori* power analysis with semPower, an R-package providing several functions to perform power analyses for structural equation models (Moshagen and Bader, 2023). A power analysis for global hypothesis testing regarding the comparison of a theoretical (hypothesized) model against the pruned model was performed to detect an effect of critical interest for the overall structure of the model. Thus, we first defined the theoretical model using lavaan syntax (requiring lavaan R-package; Rosseel, 2012), and then estimated the degrees of freedom (*df*) running the ‘semPower.getDf()’ function of the semPower package. After obtaining the model *df*, the ‘semPower.aPriori()’ function was run to determine the necessary sample size, setting the Root Mean Square Error of Approximation fit index (*RMSEA*) as the effect size measure (Jobst et al., 2023). The analysis indicated that we needed at least 193 participants to ensure detection of an effect of critical interest with a power of 0.80 at an alpha level of 0.05 with an effect size (RMSEA) of 0.06 and a resulting degree of freedom of 40.

The sample was recruited from four public schools located in Southern Italy. Participants were included in the study only if the following inclusion criteria were satisfied: (i) typical cognitive development as expressed by a score higher than the 15th percentile at the Raven’s Coloured Progressive Matrices test (CPM; Raven, 1976); (ii) lack of clinical diagnosis of neurologic, neuropsychological or neuropsychiatric disorders, as reported by parents. One hundred ninety-five fulfilled the inclusion criteria (112 females; M age = 8.6 years; SD = 1.1 year; range = 7–11 years) and were included in the analysis. All children spoke Italian as their native language, and their socioeconomic status (SES) was estimated using the Hollingshead Four Factor Index of social status status^1^, by a weighted average of education and occupational level of both parents of each child (Venuti and Senese, 2007). The sample had a mean socioeconomic status (SES) of 27.2 (SD = 14.5; range: 4.5–66). The study was conducted in accordance with the Declaration of Helsinki and approved by the Local Ethics Committee (Ethical approval code N:34/2020). Written informed consent was obtained from all parents of participants prior to testing.

### Measures

All participants underwent a formalized assessment of verbal and visuospatial working memory (Digit and Corsi span tests; Bisiacchi et al., 2005; Cornoldi and Vecchi, 2003), spatial attention (from the Spatial Abilities Test, TAS; La Femina et al., 2009), inhibition (Inhibitions test from the NEPSY-II battery; Korkman et al., 2007), verbal skills (Vocabulary subtest from the Wechsler Intelligence Scale for Children, WISC-IV; Wechsler, 2004; Naming test from the Neuropsychological evaluation battery for the developmental age, BVN; Gugliotta et al., 2009), figure disembedding (from TAS, La Femina et al., 2009; Gottschaldt’s Hidden Figure Test, GHFT, Capitani et al., 1988), mental rotation (from TAS; La Femina et al., 2009) and visual-motor coordination (from the Developmental Test of Visual-Motor Integration, VMI; Beery, 1995). The Rey-Osterrieth Complex Figure copying test (ROCF; Rey, 1983) and Block Design subtest (BD; from WISC-IV; Wechsler, 2004) were administered as measures of spatial construction.

#### Verbal working memory (Digit-F and Digit-B)

The Digit span test (Bisiacchi et al., 2005) was used to assess the passive (digit span forward) and the active (digit span backward) component of verbal working memory (Baddeley, 1986). Children were required to verbally reproduce numerical sequences of increasing length in the exact (digit span backward; Digit-F) or reverse (digit span backward; Digit-B) order with respect to the presentation order provided by the examiner. For both versions of the test, the total score was computed (score range: 0–9), with higher scores indicating a higher ability.

#### Spatial working memory (Corsi-F and Corsi-B)

The Corsi span test (Cornoldi and Vecchi, 2003; Spinnler and Tognoni, 1987) was used to assess the passive (Corsi forward) and the active (Corsi backward) component of spatial working memory (Baddeley, 1986). Children were required to reproduce block-tapping sequences of increasing length in the exact (Corsi forward; Corsi-F) or reverse (Corsi backward; Corsi-B) order with respect to the presentation order provided by the examiner. For both versions of the test, the total score was computed (score range: 0–9), with higher scores indicating a higher ability.

#### Spatial attention (SA)

Spatial attention was evaluated by means of the “visual analysis” section from the TAS battery (La Femina et al., 2009). The section comprises four visual search tasks. The initial two tasks assess visual exploration, i.e., the ability to detect all stimuli presented in the spatial field. The remaining two tasks assess selective attention, i.e., the ability to detect a specific target (triangle) while ignoring irrelevant stimuli (other geometric shapes). Based on Senese et al.’ (2020) study, we considered latency of the performance as an index of spatial attention. To get a single measure of spatial attention, a mean score (seconds) was calculated for the four tasks, with higher scores indicating a lower ability.

#### Inhibition (IN)

Inhibition was evaluated by two conditions of the Inhibition test from the NEPSY-II (Urgesi et al., 2011; Korkman et al., 2007). In both conditions, a series of black and white geometrical figures (circles or squares) or arrows were presented to participants. In Condition B, children had to respond “square” to the presentation of a circle and vice versa or respond “up” to the presentation of an arrow pointing down and vice versa, inhibiting the automatic response. In Condition C, children were required to correctly name the geometrical shapes (or the direction of arrows) printed in black while providing the opposite response to shapes (or arrows) printed in white. In both conditions, each incorrect response is scored 1 (score range: 0–40). To get a single measure of inhibition, a mean score was calculated for the two measures, with higher scores indicating a lower ability.

#### Verbal abilities (VOCAB and NAMING)

Verbal abilities were assessed by means of the Vocabulary subtest from the Wechsler Intelligence Scale for Children (WISC-IV; Orsini et al., 2012; Wechsler, 2004) and the Naming task from BVN battery (Gugliotta et al., 2009). The Vocabulary subtest is composed of 36 items, including 4 picture items, in which participants name pictures illustrated in the Stimulus Book, and 32 verbal items, in which participants have to provide definitions for words pronounced by the examiner. For each picture item, a correct choice is scored 1, while for verbal items scores range from 0 to 2. The total score ranged from 0 to 68, with higher scores indicating a higher ability.

The Naming task from BVN battery (Gugliotta et al., 2009) requires participants to name a series of images, e.g., depicting objects or animals, without any time restriction. Each correct response is scored 1. The total score ranged from 0 to 88, with higher scores indicating a higher ability.

#### Figure disembedding (FD)

The assessment of figure disembedding comprised two tasks. In both cases, participants were required to mentally disassemble the target stimulus to provide the correct response. In the Hidden Figure test (HF; La Femina et al., 2009; Trojano et al., 2015) participants had to detect the figure embedded in the target stimulus among six distracters. The task is composed of 12 items of increasing complexity, and each correct choice is scored 1 (score range: 0–12). The Gottschaldt’s Hidden Figure Test (GHFT; Capitani et al., 1988; Conson et al., 2022) requires participants to identify and highlight simple shapes embedded within complex geometric figures. The task is composed of 34 items, and each correct choice is scored 1 (score range: 0–34). To get a single measure of figure disembedding, a mean accuracy score was calculated for the two measures, with higher scores indicating a higher ability.

#### Mental rotation (MR)

Mental rotation abilities were evaluated by means of the Mental rotation task from the TAS battery (MR; La Femina et al., 2009; Trojano et al., 2015). The task requires participants to identify the item matching the target stimulus after a mental rotation among six options. The shapes can be rotated on the horizontal plane by 45°, 90°, 135°, or 180°. The task is composed of 9 items of increasing complexity; each correct choice is scored 1 (score range: 0–9), with higher scores indicating a higher ability.

#### Visual-motor coordination (MOTOR)

The visual-motor coordination was evaluated by the Motor test of the Developmental Test of Visual-Motor Integration (VMI; Beery, 1995). The task is composed of 27 items: for the initial three items, participants were required to reproduce the previously demonstrated drawing (e.g., a circle); for items from 4 to 18, participants were required to trace a line connecting the black dot to the grey ones within the shape targets, ensuring that they did not exceed the margins; for items from 19 to 27, the shapes did not contain any dots and participants were required to draw lines within the borders, in accordance with the small exemplar drawing placed above each stimulus (see also Gerth et al., 2016). After performing the first three items, participants were allowed five minutes to complete the task. Accuracy scores are assigned adopting the Beery’s (1995) scoring system, and each correct choice was scored 1 (score range: 0–27), with higher scores indicating a higher ability.

#### Rey-Osterrieth complex figure (ROCF)

Drawing was assessed by means of the immediate copying of the ROCF (Osterrieth, 1944; Rey, 1941, 1983; Conson et al., 2019). The task requires participant to copy a complex drawing made up of 18 geometrical elements, without any time restriction. Accuracy scores are assigned adopting the Rey’s (1983) scoring system, assigning for each graphic element from 0 to 2 points: 2 points when the element is completely and properly placed; 1 point when it is incomplete but properly placed, or when is complete but poorly placed; 0.5 points when the element is incomplete and poorly placed but recognisable; 0 points when it is absent or not recognisable. The total score ranged from 0 to 36, with higher scores indicating a higher ability.

#### Block design (BD)

Block building was assessed by means of the Block Design subtest from the Italian adaptation of Wechsler Intelligence Scale for Children (WISC-IV; Orsini et al., 2012; Wechsler, 2004). The task requires participants to observe bi- or three-dimensional models and use red and white blocks to reproduce them within a time limit.

The task is composed of 14 items, and the total time needed to solve each item was recorded. Items had a different scoring system: for the initial three items, each correct choice in first presentation and within the time limit is scored 2, while in second presentation is scored 1; for the items 4-to-8, each correct choice within time limit is scored 4, while, for the items 9-to-14, each correct choice is scored in relation to the time taken to respond (i.e., 7 points for a correct choice within the time range of 1–30 s, 6 points for a correct choice within the time range of 31–50 s, 5 points for a correct choice within the time range of 51–70 s and 4 points for a correct choice within the time range of 71–120 s). The total score ranged from 0 to 68, with higher scores indicating a higher ability.

### Procedure

The participants were recruited through advertisements posted in public schools in southern Italy. Following signing of the written informed consent, parents were asked to complete a personal data form, which included various information about their child (sex, age, native language, and anamnestic data on past and current psychiatric, neurological, and neurodevelopmental conditions) and a questionnaire on socioeconomic status (SES; Venuti and Senese, 2007). Each child was individually tested in a quiet room at school over two sessions, each lasting about 30 min. The administration order of the tests was randomized across participants. Prior to starting, children were informed that they had the option to stop testing at any time, but this never occurred during the data collection.

### Statistical analysis

Preliminary descriptive analyses were carried out to examine missing values and variables distributions. Univariate distributions of observed variables were evaluated for normality (Tabachnick and Fidell, 1996). Absolute skewness value ≤2 and absolute kurtosis value ≤4 were used as reference values to determine normality of distributions (Kim, 2013; Mishra et al., 2019). Then, a path analysis was executed to test the direct and indirect (mediated) effect of exogenus (sex, age and socioeconomic status) and endogenus (verbal and spatial working memory, spatial attention, inhibition, verbal abilities, figure disembedding, mental rotation and visual-motor coordination) variables on the two spatial construction measures (ROCF and BD). Path analysis is a multivariate technique which tests theoretical relations among multiple observable variables (Kline, 2011). According to the assumed theoretical model, to identify which paths to include in the basic model and to exclude non-significant paths, a bivariate correlation analysis was executed. Specifically, the correlation analysis was only performed for descriptive purposes to initially identify which paths to include in the basic model, and the Pearson correlation coefficients (zero-order correlations) between the variables were considered as an exploratory, data-driven analysis to define the basic model. Consequently, the basic model included all paths between the variables that showed a significant association based on the correlation analysis (i.e., zero-order correlations with a *p*-value <0.05). Once the basic model was defined, the first analysis was carried out to check the fit to the data. Given the specificity of the path analysis, to define potentially significant paths to add, that were not initially detected by the zero-order correlations, modification indexes (*MI*; Kline, 2011) of the tested model were also considered. The model including all the relevant paths was considered as the reference model. Then, the non-significant paths were removed, and the pruned model was compared with the reference model to verify that the more parsimonious model did not cause a significant reduction in the fit. Once identified the most parsimonious model, to explore the stability and generalizability of the model, the invariance of the model parameters was tested between male and female groups (for a detailed description of the procedure, see Zappullo et al., 2023a,b). Given that path analysis is not a fully appropriate statistical approach for a sample size below 100 observations (Kline, 2011), it is noted that the invariance analysis between sexes should be considered as exploratory. In all analyses, path coefficients were estimated with LISREL 8.71 software (Jöreskog and Sörbom, 2004) and the maximum likelihood method, based on covariance matrices. As fit indices, we used the Maximum Likelihood (*MLχ*^2^) goodness-of-fit test statistics in combination with Root Mean Square Error of Approximation index (*RMSEA*); Normed Fit Index (*NFI*); Comparative Fit Index (*CFI*); Goodness-of-Fit statistic (*GFI*); and the ratio *MLχ*^2^/*df* (Cheung and Rensvold, 2002; Kline, 2011). The following values were considered as indicating good fitting models: *p* > 0.05 for *MLχ*^2^ test; values ≤0.08 for *RMSEA*; values ≥0.95 for *NFI*; values >0.90 for *CFI*; values ≥0.95 for GFI; values <3 for ratio *MLχ*^2^/*df*.

## Results

Preliminary descriptive analyses showed no missing values or violations of normality assumption (Table 1). Pearson’s correlation coefficients (zero-order correlations) between all the considered variables are reported in Table 2. All the significant zero-order correlations (all *p* < 0.05) were considered in defining the paths of the basic model.

**Table 1:** Descriptive analysis of the variables of interest.

Variables^a^	*M*	SD	Min	Max	Skewness	Kurtosis
1. DIGIT-F	4.43	0.71	3	7	0.73	0.50
2. DIGIT-B	3.07	0.90	0	6	0.48	0.81
3. CORSI-F	4.17	0.87	2	7	0.64	0.80
4. CORSI-B	3.63	1.10	2	6	0.25	−0.72
5. SA	59.50	17.30	30.5	129.5	1.10	1.37
6. IN	7.75	4.78	0.5	28.5	1.63	3.94
7. NAMING	50.45	6.93	25	68	−0.40	0.54
8. VOCAB	29.62	7.63	15	48	0.29	−0.70
9. FD	12.96	4.34	1.5	23	−0.52	−0.05
10. MR	2.40	1.96	0	9	1.11	1.09
11. MOTOR	19.03	3.65	7	26	−0.61	0.42
12. ROCF	21.70	5.67	8.5	35	0.06	−0.40
13. BD	20.69	9.65	3	45	0.33	−0.45

*N* = 195.

a Digit-F, forward digit span test; Digit-B, backward digit span test; Corsi-F, forward Corsi span test; Corsi-B, backward Corsi span test; SA, Speed of Visual search tasks from TAS; IN, Errors of Inhibition and Switching conditions of Inhibition Test from NEPSY-II; NAMING, Naming task from BVN; VOCAB, Vocabulary subtest from WISC-IV; FD, composite index for figure disembbeding (Hidden figure identification TAS battery and modified version of GHFT); MR, mental rotation (TAS battery); MOTOR, visual-motor coordination from VMI battery; ROCF, copying of the Rey-Osterrieth Complex Figure; BD, block design subtest from WISC-IV.

**Table 2:** Intercorrelations between variables.

Variables^a^	1	2	3	4	5	6	7	8	9	10	11	12	13	14	15	16
1. SEX	–															
2. AGE	0.067	–														
3. SES	0.087	−0.088	–													
4. DIGIT-F	0.075	0.292***	0.168*	–												
5. DIGIT-B	0.062	0.448***	0.126	0.282***	–											
6. CORSI-F	0.082	0.218**	0.166*	0.287***	0.324***	–										
7. CORSI-B	−0.044	0.340***	0.161*	0.205**	0.376***	0.376***	–									
8. SA	−0.129	−0.411***	−0.162*	−0.314***	−0.186**	−0.193**	−0.163*	–								
9. IN	0.025	−0.285***	−0.007	−0.186**	−0.287***	−0.125	−0.263***	0.171*	–							
10. NAMING	0.200**	0.444***	0.171*	0.224**	0.428***	0.258***	0.265***	−0.115	−0.362***	–						
11. VOCAB	0.058	0.613***	0.076	0.362***	0.385***	0.227***	0.353***	−0.422***	−0.356***	0.551***	–					
12. FD	0.136	0.515***	0.136	0.346***	0.410***	0.419***	0.433***	−0.227**	−0.367***	0.416***	0.430***	–				
13. MR	−0.034	0.294***	0.276***	0.240***	0.308***	0.316***	0.265***	−0.101	−0.171*	0.334***	0.347***	0.434***	–			
14. MOTOR	0.057	0.431***	−0.096	0.153*	0.155*	0.298***	0.263***	−0.117	−0.179*	0.265***	0.390***	0.445***	0.231**	–		
15. ROCF	0.110	0.464***	0.048	0.322***	0.342***	0.290***	0.302***	−0.342***	−0.364***	0.310***	0.440***	0.596***	0.296***	0.432***	–	
16. BD	−0.017	0.498***	0.132	0.324***	0.392***	0.401***	0.496***	−0.181*	−0.299***	0.419***	0.494***	0.693***	0.479***	0.445***	0.449***	–

*N* = 195. *p < 0.05; **p < 0.01; ***p < 0.001.

a SEX, participants’ sex (dummy coding, males = 0, females = 1); SES, family socioeconomic status; Digit-F, forward digit span test; Digit-B, backward digit span test; Corsi-F, forward Corsi span test; Corsi-B, backward Corsi span test; SA, Speed of Visual search tasks from TAS; IN, Errors of Inhibition and Switching conditions of Inhibition Test from NEPSY-II; NAMING, Naming task from BVN; VOCAB, Vocabulary subtest from WISC-IV; FD, composite index for figure disembbeding (Hidden figure identification TAS battery and modified version of GHFT); MR, mental rotation (TAS battery); MOTOR, visual-motor coordination from VMI battery; ROCF, copying of the Rey-Osterrieth Complex Figure; BD, block design subtest from WISC-IV.

**Figure 1:**
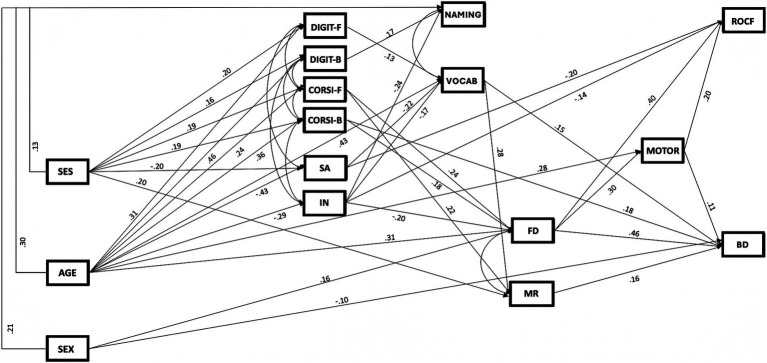
Path diagram for the best fitting model predicting drawing performance (*N* = 195). Paths were represented with arrows and non-directional correlations with arcs (all *p* < 0.05). Each arrow was associated to a standardized coefficient. SEX, participants’ sex (dummy coding: males = 0, females = 1); SES, family socioeconomic status; Digit-F, forward digit span test; Digit-B, backward digit span test; Corsi-F, forward Corsi span test; Corsi-B, backward Corsi span test; SA, Speed of Visual search tasks from TAS; IN, Errors of Inhibition and Switching conditions of Inhibition Test from NEPSY-II; NAMING, Naming task from BVN; VOCAB, Vocabulary subtest from WISC-IV; FD, composite index for figure disembbeding (Hidden figure identification TAS battery and modified version of GHFT); MR, mental rotation (TAS battery); MOTOR, visual-motor coordination from VMI battery; ROCF, copying of the Rey-Osterrieth Complex Figure; BD, block design subtest from WISC-IV. *N* = 195.

The first path analysis carried out on the total sample showed an acceptable fit for the basic model, *MLχ*^2^(26) = 52.26; *p* = 0.0016; *RMSEA* = 0.073; *CFI* = 0.99. The analysis of *MI*s indicated that the inclusion of paths between SEX and figure disembedding (FD), SEX and block design (BD), SEX and mental rotation (MR) and between socioeconomic status (SES) and digit span forward (DIGIT-F) for gamma matrix, would have improved the fit of the model. Therefore, these additional paths were included in the basic model, that was considered as the new basic model and the fit of the model was tested again. Results of the path analysis showed a good fit for the corrected basic model that considered all the significant paths between variables, *MLχ*^2^(22) = 29.29; *p* = 0.136; *MLχ^2^/df* = 1.33; *RMSEA* = 0.042, 90% CI [0.00–0.078]; *ECVI* = 1.35; *NFI* = 0.99; *CFI* = 1.00; *GFI* = 0.98; *AIC* = 257.29; *CAIC* = 744.41. Subsequently, the non-significant paths were pruned (all *p* > 0.05), and the fit of the pruned model was tested, showing again a good fit for the model, *MLχ*^2^(71) = 84.34; *p* = 0.133; *MLχ^2^/df* = 1.18; *RMSEA* = 0.031, 90% CI [0.00; 0.055]; *ECVI* = 1.12; *NFI* = 0.96; *CFI* = 0.99; *GFI* = 0.95; *AIC* = 214.34; *CAIC* = 492.09, and that the more parsimonious model did not cause a significant reduction in the fit, *MLχ*^2^_diff_ (49) = 55.05, *p* = 0.256; *CFI*_diff_ = 0.01. This latter model was considered the best fitting model (Figure 1).

As shown in Figure 1, as regards the drawing performance, data showed that the ROCF score was directly related with spatial attention (SA), inhibition (IN), figure disembedding (FD) and visual-motor coordination (MOTOR), whereas indirectly related with SEX (*p* = 0.005), AGE (*p* < 0.001), socioeconomic status (SES; *p* < 0.001), Corsi span forward (CORSI-F; *p* < 0.001) and backward (CORSI-B; *p* = 0.004), inhibition (IN; *p* = 0.001) and figure disembedding (FD; *p* = 0.009). Instead, the Block Design (BD) score was directly related with SEX, Corsi span backward (CORSI-B), vocabulary (VOCAB), figure disembedding (FD), mental rotation (MR) and visual-motor coordination (MOTOR), and indirectly related with SEX (*p* = 0.004), AGE (*p* < 0.001), socioeconomic status (SES; *p* < 0.001), digit span forward (DIGIT-F; *p* = 0.035), Corsi span forward (CORSI-F; *p* < 0.001) and backward (CORSI-B; *p* = 0.004), spatial attention (SA; *p* = 0.006), inhibition (IN; *p* < 0.001) and vocabulary (VOCAB; *p* = 0.013).

Moreover, data showed that: (i) visual-motor coordination (MOTOR) was directly related with figure disembedding (FD) and indirectly with SEX (*p* = 0.015), AGE (*p* < 0.001), Corsi span forward (CORSI-F; *p* = 0.003) and backward (CORSI-B; *p* = 0.013) and inhibition (IN; *p* = 0.006); (ii) mental rotation (MR) was directly related with Corsi span forward (CORSI-F) and vocabulary (VOCAB), and indirectly with AGE (*p* < 0.001), socioeconomic status (SES; *p* = 0.003), digit span forward (DIGIT-F; *p* = 0.03), spatial attention (SA; *p* = 0.003) and inhibition (IN; *p* = 0.012); (iii) figure disembedding (FD) was directly related with Corsi span forward (CORSI-F) and backward (CORSI-B) and inhibition (IN) and indirectly with AGE (*p* < 0.001) and socioeconomic status (SES; *p* = 0.002); (iv) vocabulary (VOCAB) were directly related with digit span forward (DIGIT-F), spatial attention (SA) and inhibition (IN) and indirectly with AGE (*p* < 0.001) and socioeconomic status (SES; *p* = 0.002); (v) naming (NAMING) was directly related with digit span backward (DIGIT-B) and inhibition (IN) and indirectly with AGE (*p* < 0.001).

As regards the exogenous variables, (i) SEX had a direct effect on naming (NAMING), figure disembedding (FD) and block design (BD); (ii) AGE had a direct effect on digit span forward (DIGIT-F) and backward (DIGIT-B), Corsi span forward (DIGIT-F) and backward (DIGIT-B), spatial attention (SA), inhibition (IN), naming (NAMING) and vocabulary (VOCAB), figure disembedding (FD) and visual-motor coordination (MOTOR); (iii) socioeconomic status (SES) had a direct effect on digit span forward (DIGIT-F) and backward (DIGIT-B), Corsi span forward (DIGIT-F) and backward (DIGIT-B), spatial attention (SA), naming (NAMING) and mental rotation (MR). Standardized direct and indirect effects are also reported in Supplementary Tables S1, S2, respectively.

In synthesis, the results showed that all considered variables had a relevant effect on spatial construction, with distinct patterns for the two considered dependent measures. Moreover, data showed that the considered variables accounted for a relevant percentage of the variance of the spatial construction measures: 45% of the variance of ROCF and 57% of the variance of the BD. Furthermore, they also accounted for a sizeable proportion of variance of other measures: digit span forward (DIGIT-F: 12%) and backward (DIGIT-B: 23%), Corsi span forward (DIGIT-F: 8%) and backward (DIGIT-B: 15%), spatial attention (SA: 21%), inhibition (IN: 8%), naming (NAMING: 36%) and vocabulary (VOCAB: 46%), figure disembedding (FD: 43%), mental rotation (MR: 20%) and visual-motor coordination (MOTOR: 25%).

### Invariance analysis

We first evaluated the configural invariance (invariance of model form). To this aim, a simultaneous multi-group path model of covariance structure was tested in males and females. This model imposes no equality constraints on parameter estimates across groups. Results indicated an acceptable fit for the tested model, *MLχ*^2^(118) = 142.06; *p* = 0.065; *RMSEA* = 0.065, *CFI* = 0.98. Therefore, to test the invariance of the model, the same path model was tested simultaneously in both sexes but constraining the corresponding path coefficients to be equal in the two groups. Results showed a low fit for the invariant model, *MLχ*^2^(179) = 229.75; *p* = 0.006; *RMSEA* = 0.054, *CFI* = 0.97, and those constraints caused a significant reduction in fit, *MLχ*^2^_diff_ (61) = 87.69, *p* = 0.014; *CFI*_diff_ = 0.01. The *MIs* and the parameter estimate analysis suggested freely estimating the paths of Gamma matrix. The new partial invariance model showed an acceptable fit to the data, *MLχ*^2^(162) = 191.51; *p* = 0.056; *RMSEA* = 0.044, *CFI* = 0.98, and a non-significant loss of fit compared to the configural model, *MLχ*^2^_diff_ (44) = 49.45, *p* = 0.264; *CFI*_diff_ = 0.00.

### Summary of the results

The main results showed that figure disembedding (FD) and visual-motor coordination (MOTOR) were the only shared predictors of ROCF copying and Block Design, while there was a distinct pattern of predictors specifically and directly associated with the two spatial construction tests. ROCF was specifically related with inhibition (IN) and spatial attention (SA), whereas Block Design (BD) was specifically related with sex, Corsi span backward (CORSI-B), vocabulary (VOCAB) and mental rotation (MR). Moreover, the model showed a partial sex invariance, since it maintained the same path structure between the sexes but showed a difference in parameter estimation for paths involving AGE and SES (gamma matrix).

## Discussion

The present data showed that figure disembedding and visual-motor coordination were the only shared predictors of ROCF copying and Block Design. These results confirm that the efficiency of global/local visual processing plays an important role in drawing performance (De Renzi, 1982; Del Giudice et al., 2000; La Femina et al., 2009; Zappullo et al., 2020, 2021). More relevant, these results revealed that visual-motor coordination is important to copying tasks, regardless of whether the to-be-reproduced model needs to be drawn or assembled, consistent with data demonstrating, on one hand, a positive association between eye-hand coordination and both drawing (Anderson et al., 2001; Dilworth et al., 2004; Weber et al., 2013) and block building (Jeon et al., 2013), and, on the other hand, a poor performance on Block Design in people with poor motor coordination abilities (Jascenoka and Walter, 2022).

The co-occurrence of the direct effects of figure disembedding and visual-motor coordination on both spatial construction measures might suggest that, as in drawing (Chamberlain and Wagemans, 2016; Senese et al., 2020), even in the case of Block Design, figure disembedding would guide building the mental representation of the to-be-reproduced model, thus enabling the activation of the visual-motor stage. This interpretation would also be supported by the direct relation we found between figure disembedding and visual-motor coordination.

Our results also demonstrated effects that were specific for each of the two tasks. Indeed, ROCF performance was specifically and directly related with spatial attention and inhibition while Block Design was specifically and directly related with sex, vocabulary, mental rotation and spatial working memory (Corsi span backward). Regarding the effect of spatial attention on ROCF, it is in line with previous studies demonstrating both direct (Senese et al., 2015) and mediated (Senese et al., 2020) effects of attention on this test. In the same vein, the direct and indirect relationships between inhibition and drawing found here is partially consistent with results from a previous path model showing that figure disembedding mediated the effect of inhibition on ROCF (Zappullo et al., 2021).

As for the Block Design performance, the present results provided further support to the specific contribution of sex, verbal abilities and mental rotation to block building (Giofrè et al., 2022a,b, 2023; Zappullo et al., 2021), and, more importantly, revealed the contribution of spatial working memory. Although a positive relationship between working memory and Block Design performance has been suggested in typically developing children (Rahbari and Vaillancourt, 2015), the nature of such relationship remains to be elucidated. Adult neuropsychological studies on three-dimensional block building suggest that since this task is functionally serialized into specific sequential sub-goals that add up to a correct final copy, spatial working memory would allow both the final goal and a set of sub-subgoals to be maintained, enabling online comparison between one’s own results and the desired outcome during the spatial construction process (Ballard et al., 1992, 1997; Shelton et al., 2022). Although three-dimensional block building and Block Design cannot be considered equivalent, they shared some key aspects. In both cases, the “spatial problem” requires the physical manipulation, rotation and combination of the blocks by relying on the analysis of the spatial relationships between them (Casey et al., 2008). Therefore, it could be speculated that, as for three-dimensional building, even in the case of Block Design the active component of spatial working memory would maintain both the single elements and the final configuration actively processed, supporting the on-going building-up of a coherent spatial representation of the whole figure to enable step-by-step actions (Ballard et al., 1992, 1997).

The presence of specific patterns of cognitive predictors of ROCF and Block Design may suggest the idea that they elicit distinct reproduction strategies. As recalled above, drawing could imply the activation of a strategy in which spatial attention (through continuous eye movements between the copy and the model) contributes to build a mental representation of the figure and activates the visuo-motor transformation, guiding the hand on the sheet, with a lower load on working memory (Tchalenko and Miall, 2009; Trojano et al., 2009; Chamberlain and Wagemans, 2016). In such a strategy, the inhibitory control could contribute to the monitoring process, supporting an online representation of the figure (Zappullo et al., 2021). Instead, during block building a different, sequential strategy could be activated in which spatial working memory maintains in parallel both the local elements and the global model to be reproduced, supporting building-up a coherent spatial representation of the whole figure (Shelton et al., 2022; Ballard et al., 1997; Casey et al., 2008). Once the mental representation of the image has been created, mental rotation abilities would allow mental manipulation of the blocks, which must first be rotated mentally and then physically through visual-motor coordination skills (Casey et al., 2008).

Finally, the partial sex invariance for the path model, with a different weight of estimated parameters involving age and socioeconomic status, merits a comment. This finding is partially consistent with the previous observations showing a full measurement invariance between sexes (Senese et al., 2015, 2020; Zappullo et al., 2021). However, as specified above, the invariance analysis performed in the present study should be considered as exploratory, thus caution is needed in the generalizability of the results (Kline, 2011).

Some limitations also merit comment. First, we tested cognitive predictors of the two most widely used spatial construction measures, both tapping the ability to copy a geometric model. Neuropsychological dissociations have been found between copying and free drawing in adult patients with brain damage (Gainotti and Trojano, 2018). Therefore, the present findings specifically referring to copying tasks call for future investigations verifying generalizability of our path model to free drawing performance. Second, the present study adopted a correlational design. Although path analysis is useful for exploring the relationships between different variables based on the theoretical assumptions on the type and direction of the links between the variables studied, this statistical approach cannot be considered fully informative of the causal inference of the data (Kline, 2011).

Notwithstanding the above limitations, the present findings allow to discuss some clinical implications. Indeed, identifying task-specific predictors of the two spatial construction measures supports their non-equivalence in terms of neuropsychological underpinnings. This would help to prevent potential interpretative errors in the assessment of spatial construction disorders (Studeny et al., 2019; Peart and Bennett, 2024). Furthermore, the identification of skills equally involved in drawing and block building could be relevant to deal with clinical conditions in which deficits in drawing and block building tasks tend to co-occur, as in the case children with Williams Syndrome and Nonverbal Learning Disability (Van Herwegen, 2015; Mammarella and Cornoldi, 2014). In such cases of pervasive spatial construction disorders, implementing interventions focused on “core,” shared, cognitive mechanisms could represent a valuable treatment option.

## Data Availability

The raw data supporting the conclusions of this article will be made available by the authors, without undue reservation.

## Footnotes

^1^Hollingshead, A. (1975). The four-factor index of social status. Unpublished manuscript. Yale University, Department of Sociology, New Haven, CT.
