# Supplementary material for: Specific and shared cognitive predictors of drawing and block building in typically developing children
Source: Front Hum Neurosci. 2024 Sep 25;18:1436362. doi: 10.3389/fnhum.2024.1436362 (PMC11461352; doi:10.3389/fnhum.2024.1436362)
Supplement: Supplementary file 1 [file Table_1.docx]

**Table S1.** Standardized Direct Effects for Beta and Gamma matrices.

| **Variables^** | **1** | **2** | **3** | **4** | **5** | **6** | **7** | **8** | **9** | **10** | **11** | **12** | **13** | **14** | **15** | **16** |
| --- | --- | --- | --- | --- | --- | --- | --- | --- | --- | --- | --- | --- | --- | --- | --- | --- |
| **1. SEX** | - | - | - | - | - | - | - | - | - | - | - | - | - | - | - | - |
| **2. AGE** | - | - | - | - | - | - | - | - | - | - | - | - | - | - | - | - |
| **3. SES** | - | - | - | - | - | - | - | - | - | - | - | - | - | - | - | - |
| **4. DIGIT-F** | - | .31*** | .20** | - | - | - | - | - | - | - | - | - | - | - | - | - |
| **5. DIGIT-B** | - | .46*** | .16** | - | - | - | - | - | - | - | - | - | - | - | - | - |
| **6. CORSI-F** | - | .24*** | .19** | - | - | - | - | - | - | - | - | - | - | - | - | - |
| **7. CORSI-B** | - | .36*** | .19** | - | - | - | - | - | - | - | - | - | - | - | - | - |
| **8. SA** | - | -.43*** | -.20** | - | - | - | - | - | - | - | - | - | - | - | - | - |
| **9. IN** | - | -.29*** | - | - | - | - | - | - | - | - | - | - | - | - | - | - |
| **10. NAMING** | .21*** | .30*** | .13* | - | .17** | - | - | - | -.24*** | - | - | - | - | - | - | - |
| **11. VOCAB** | - | .43*** | - | .13* | - | - | - | -.22*** | -.17** | - | - | - | - | - | - | - |
| **12. FD** | .16** | .31*** | - | - | - | .24*** | .18** | - | -.20*** | - | - | - | - | - | - | - |
| **13. MR** | - | - | .20** | - | - | .22*** | - | - | - | - | .28*** | - | - | - | - | - |
| **14. MOTOR** | - | .28*** | - | - | - | - | - | - | - | - | - | .30*** | - | - | - | - |
| **15. ROCF** | - | - | - | - | - | - | - | -.20*** | -.14* | - | - | .40*** | - | .20*** | - | - |
| **16. BD** | -.10* | - | - | - | - | - | .18** | - | - | - | .15*** | .46*** | .16** | .11* | - | - |

***Note***. ^Values are expressed as standardized path coefficients (e.g., partial regression coefficients). Direct effects for Gamma (e.g., causal paths from exogenous to endogenous variables) and Beta (e.g., causal paths among endogenous variables) matrices are reported in black and blue, respectively.

SEX: participants’ sex (dummy coding: males = 0, females = 1); SES: family socioeconomic status; Digit-F: forward digit span test; Digit-B: backward digit span test; Corsi-F: forward Corsi span test; Corsi-B: backward Corsi span test; SA: Speed of Visual search tasks from TAS; IN: Errors of Inhibition and Switching conditions of Inhibition Test from NEPSY-II; NAMING: Naming task from BVN; VOCAB: Vocabulary subtest from WISC-IV; FD: composite index for figure disembbeding (Hidden figure identification TAS battery and modified version of GHFT); MR: mental rotation (TAS battery); MOTOR: visual-motor coordination from VMI battery; ROCF: copying of the Rey-Osterrieth Complex Figure; BD: block design subtest from WISC-IV. *N* = 195. **p* < .05; ***p* < .01; ****p* < .001.

**Table S2.** Standardized Indirect Effects (*SIE*) for Beta and Gamma matrices.

| **Variables^** | **1** | **2** | **3** | **4** | **5** | **6** | **7** | **8** | **9** | **10** | **11** | **12** | **13** | **14** | **15** | **16** |
| --- | --- | --- | --- | --- | --- | --- | --- | --- | --- | --- | --- | --- | --- | --- | --- | --- |
| **1. SEX** | - | - | - | - | - | - | - | - | - | - | - | - | - | - | - | - |
| **2. AGE** | - | - | - | - | - | - | - | - | - | - | - | - | - | - | - | - |
| **3. SES** | - | - | - | - | - | - | - | - | - | - | - | - | - | - | - | - |
| **4. DIGIT-F** | - | - | - | - | - | - | - | - | - | - | - | - | - | - | - | - |
| **5. DIGIT-B** | - | - | - | - | - | - | - | - | - | - | - | - | - | - | - | - |
| **6. CORSI-F** | - | - | - | - | - | - | - | - | - | - | - | - | - | - | - | - |
| **7. CORSI-B** | - | - | - | - | - | - | - | - | - | - | - | - | - | - | - | - |
| **8. SA** | - | - | - | - | - | - | - | - | - | - | - | - | - | - | - | - |
| **9. IN** | - | - | - | - | - | - | - | - | - | - | - | - | - | - | - | - |
| **10. NAMING** | - | .15*** | .03 | - | - | - | - | - | - | - | - | - | - | - | - | - |
| **11. VOCAB** | - | .18*** | .07** | - | - | - | - | - | - | - | - | - | - | - | - | - |
| **12. FD** | - | .18*** | .08** | - | - | - | - | - | - | - | - | - | - | - | - | - |
| **13. MR** | - | .22*** | .06** | .04* | - | - | - | -.06** | -.05* | - | - | - | - | - | - | - |
| **14. MOTOR** | .05* | .15*** | .02 | - | - | .07** | .06* | - | -.06** | - | - | - | - | - | - | - |
| **15. ROCF** | .07** | .41*** | .08*** | - | - | .11*** | .09** | - | -.09** | - | - | .06** | - | - | - | - |
| **16. BD** | .08** | .47*** | .12*** | .03* | - | .16*** | .09** | -.04** | -.13*** | - | .04* | .03 | - | - | - | - |

***Note***. ^Standardized Indirect effects for Gamma (e.g., causal paths from exogenous to endogenous variables) and Beta (e.g., causal paths among endogenous variables) matrices are reported in black and blue, respectively.

SEX: participants’ sex (dummy coding: males = 0, females = 1); SES: family socioeconomic status; Digit-F: forward digit span test; Digit-B: backward digit span test; Corsi-F: forward Corsi span test; Corsi-B: backward Corsi span test; SA: Speed of Visual search tasks from TAS; IN: Errors of Inhibition and Switching conditions of Inhibition Test from NEPSY-II; NAMING: Naming task from BVN; VOCAB: Vocabulary subtest from WISC-IV); FD: composite index for figure disembbeding (Hidden figure identification TAS battery and modified version of GHFT); MR: mental rotation (TAS battery); MOTOR: visual-motor coordination from VMI battery; ROCF: copying of the Rey-Osterrieth Complex Figure; BD: block design subtest from WISC-IV. *N* = 195. **p* < .05; ***p* < .01; ****p* < .001.
